# *Phytophthora polonica* and *Phytophthora hydropathica* from Clade 9 Associated with Alder Decline in Bulgaria

**DOI:** 10.3390/life14060720

**Published:** 2024-06-01

**Authors:** Petya Koeva Christova

**Affiliations:** AgroBioInstitute, Agricultural Academy, 8 Dragan Tsankov Blvd., 1164 Sofia, Bulgaria; petyachristova@abi.bg

**Keywords:** oomycetes, pathogens, rhizosphere, disease, *Alnus*

## Abstract

A number of *Phytophthora* species have been identified as destructive plant pathogens and invasive species. They have the potential to affect a wide range of host plants and cause diseases in agricultural and forest ecosystems. Two *Phytophthora* species from rhizosphere soil samples collected from declining *Alnus glutinosa* in Bulgaria were isolated in the autumn of 2022. They were identified as *Phytophthora polonica* and *Phytophthora hydropathica* according to the DNA sequence analysis of the ITS region, as well as their morphological and physiological characteristics. The pathogenicity of both species to common and gray alder was evaluated by the inoculation of detached leaves and cuttings. Experimental data proved that *P. polonica* and *P. hydropathica* are able to cause leaf necrosis not only on *A. glutinosa* from which they were derived, but also on *A. incana*. No significant deference in the aggressiveness of the studied isolates from both *Phytophthora* species against the two tested plants was observed. Therefore, *P. polonica* and *P. hydropathica* were determined as potential threats for alder ecosystems in the country. This is the first report for the isolation of *P. polonica* in Bulgaria and represents the most southeastern point of the species distribution in Europe.

## 1. Introduction

In the last 20 years, the number of new *Phytophthora* species has been growing continuously. Members of the genus are classified into 12 main clades, and some of them are divided into subclades based on molecular phylogenetic analysis [1]. Most of the *Phytophthora* species are characterized by soilborne or waterborne existences. The representatives of the genus that are associated with water habitats are mainly saprophytes. However, many *Phytophthora* species are necrotrophic or hemibiotrophic pathogens and are able to cause root rot diseases on a variety of plant hosts [2]. Some of the most devastating plant pathogens worldwide belong to the genus *Phytophthora*, including *P. infestance* [3], *P. alni* [4] and *P. ramorum* [5].

Clade 9 is among the most rapidly expanding and dynamic phylogenetic groups of the genus *Phytophthora*. In addition to previously reported species from the clade such as *P. insolita* [6], *P. macrochlamydospora* [7], *P. captiosa*, *P. fallax* [8], *P. polonica* [9], *P. irrigate* [10] and *P. parsiana* [11], several new species were described in the last years, including *P. hydropathica* [12], *P. arenaria*, *P. constricta* [13], *P. virginiana* [14], *P. stricta*, *P. macilentosa* [15], *P. hydrogena* [16] and *P. pseudopolonica* [17]. The members of clade 9 are characterized by diversity in terms of their origins, and some of them are associated with the decline of host plants, whereas the others have been derived from aquatic ecosystems.

A specific feature of the *Phytophthora* species from clade 9 is their morphological, physiological and phylogenetical diversity. Typical for the members from this group is the production of non-papillate sporangia, but there are a few species with semi-papillate sporangia as *P. macrochlamydospora* [7] and *P. constricta* [13]. Usually, sporangia are non-caducous; however, sometimes they could be slightly caducous [13]. This clade includes species that are homothallic, heterothallic or sterile; however, the isolates that require a pairing for their sexual reproduction process are always A1 mating type.

A unique characteristic of clade 9 is the ability of the species to grow at high temperatures, which is not observed for any other *Phytophthora* clade. Most of the species in this cluster tolerate temperatures of about 35 °C and demonstrate maximum growth at 40 °C. This group includes *P. insolita* [6], *P. polonica* [9], *P. irrigata* [10], *P. hydropathica* [12], *P. aquimorbida* [18], *P. virginiana* [14], *P. macilentosa* [15] and *P. hydrogena* [16]. A relatively small part of the members from the clade 9 have a maximum growth rate of about 30 °C, like *P. macrochlamydospora* [7], *P. constricta* [13] and *P. captiosa* [8].

The most devastating plant pathogen on alder is the *P. alni* complex, which includes three taxa: *P.* × *alni*, *P. uniformis* and *P.* × *multiformis* [19,20,21]. The first of them is described as the most aggressive to alder species, whereas the other two are less pathogenic and rarely isolated in Europe [4,22,23,24]. A number of studies on the disease symptoms of *Alnus* spp. revealed that the causal agent usually is not only one species but an assembly of *Phytophthora* pathogens [2,20,25,26,27,28,29]. Therefore, numerous *Phytophthora* species are associated with the declining of alder, including *P. plurivora*, *P. gonapodyides*, *P. lacustris*, *P. cactorum*, *P. syringae*, *P. pseudosyringae*, *P. polonica*, *P. hydropathica*, *P. bilorbang*, *P. acerina*, *P. siskiyouensis*, *P. pseudocryptogea*, *P.* × *serendipita* and *P. alpine*. However, studies of alder are mainly focused on *A. glutinosa* [2,20,23,24,26,30,31] and rarely concern *A. incana* [25,27,32,33]. Although several investigations on the health status of alder ecosystems in Europe have been reported recent years, the occurrence, diversity and new distribution sites of the *Phytophthora* species affecting *Alnus* spp. across the continent need further exploration.

Here, the isolation and characterization of *P. polonica* and *P. hydropathica* from the rhizosphere soil of declining common alder in southeast Bulgaria are presented. Both species belong to clade 9 and demonstrate typically for the group’s morphological and physiological features. They were determined to be pathogens not only on *A. glutinosa* but also on *A. incana* according to the results of conducted pathogenicity tests.

## 2. Materials and Methods

### 2.1. Sampling, Isolation and Cultivation of Phytophthora Species

Sampling was performed in the southeastern part of Bulgaria (Global Positioning System data point—42.060125, 27.966534) in 2022. The soil samples were collected from black alder trees (*Alnus glutinosa*) with disease symptoms, characterized by crown defoliation and dieback of twigs and branches but the absence of bleeding stem. The rhizosphere soil together with thin roots were derived at a depth of 30 cm after removing the surface soil layer along with fallen leaves and other vegetation. The soil samples (about 500 g) were stored into labeled plastic bags and transported to the laboratory.

Putative *Phytophthora* isolates were derived by using the baiting method. Each soil sample was placed in a container with a volume of about 2 L and dissolved in sterile distilled water, which covered the soil surface with an aqueous layer (3–4 cm in depth). After settling, young leaves of *Rhododendron*, oak and oleander were placed on the water’s surface. The baits were incubated for a period of 5 days at room temperature. When the symptoms of infection by putative oomycete were observed, the leaves were surface sterilized by rinsing them under running water, treating with 70% ethanol for 30 s washing three times in sterile distilled water and then drying on sterile filter paper. Small segments of the zone between the healthy and necrotic tissue were cut off and were incubated on a selective medium PARNHB (1 L vegetable agar, 10 mg Pimaricin, 250 mg Ampicillin, 10 mg Rifampicin, 50 mg Nystatin, 1.3 mL Tahigaren and 15 mg Benomyl) at the room temperature (20–25 °C). After the appearance of hyphae around the plant segments, the mycelium was transferred to water agar for isolation of a pure culture. The selected isolates were incubated on carrot agar (CA; 16 g agar, 3 g CaCO_3_, 100 mL carrot juice/1 L), vegetable agar (V8A; 16 g agar, 3 g CaCO_3_, 100 mL V8 juice/1 L) and potato dextrose agar (PDA, Difco^®^; NJ, USA) for future investigations.

### 2.2. Molecular Identification

The DNA was isolated from the mycelia of 10-day-old mycelial cultures using a DNeasy Plant Mini Kit (QIAGEN GmbH; Hilden, Germany). The PCR amplification of the internal transcribed spacer (ITS) region was performed with primers ITS5 (5′-GGAAGTAAAAGTCGTAACAAGG-3′) and ITS4 (5′-TCCTCCGCTTATTGATATGC-3′) using the following PCR program: 96 °C—2 min, followed by 35 cycles of 96 °C—1 min, 55 °C—1 min, 72 °C—2 min and final elongation at 72 °C—10 min. PCR analyses were carried out by PuReTaq^TM^ Ready-To-Go^TM^ PCR beads (GE Healthcare Life Sciences; Wien, Austria) according to the manufacturer’s instructions. The PCR products were purified using Sephedex (around 34 mg per well) that was poured into 96-well microtiter plates and flooded with dH_2_O (300 µL in each well). After incubation at room temperature for 4 h, the plates were centrifuged at 750× *g* for 90 s, and the PCR reactions (25 µL) were loaded in the wells, followed by centrifugation at the same conditions as above. The purified PCR products were sequenced at Eurofins Genomics, Germany. Species identification was performed by a search for high homology sequences of ITS region within the National Center for Biotechnology Information (NCBI) database using the Basic Local Alignment Search Tool (BLAST). Sequence alignments and phylogenetic trees of the ITS region were conducted by the MEGA7 program [34].

### 2.3. Morphological Characteristics and Radial Growth Rate

The studied *Phytophthora* isolates were cultivated on CA, V8A and PDA media at 20 °C for the characterization of colony morphology. The microscopic observation of morphological structures was performed under microscope at ×400 magnification (ZEISS Axio Imager A2; Carl Zeiss Micriscopy, Jena, Germany) with a digital camera (AxioCamERs 5S; Carl Zeiss Micriscopy, Jena, Germany) and biometric software (AxioVision LE; Carl Zeiss Micriscopy, Jena, Germany).

Different protocols for inducing sporangia formation by *P. polonica* and *P. hydropathica* were applied. The sporangia of both *P. hydropathica* isolates were produced from the agar plugs (10 × 10 mm) of 5-day-old V8A mycelial cultures that were flooded with non-sterile spring water. They were incubated into Petri dishes at natural daylight and ambient temperature for 24–48 h. However, the application of this standard and simple procedure was not successful for the production of sporangia by *P. polonica* isolates, and neither was the method described by Belbahri et al. [9]. Several different protocols were tested and an effective procedure was finally established. Agar plugs (10 × 10 mm) from the 5-day-old V8A mycelial cultures of the *P. polonica* isolates were flooded with non-sterile spring water into Petri dishes and were incubated at 5 °C. After 2 days, they were transferred at natural daylight and ambient temperature for 24–30 h.

The isolates were incubated on V8A and CA media at 20 °C for observation of different morphological structures such as chlamydospores and hyphal swelling. The formation of oogonia by *P. polonica* on CA was directly monitored, while the *P. hydropathica* isolates needed induction by the compatibility type of *P. nicotianae*. Mycelial plugs (10 × 10 mm) from the 5-day-old cultures of each of the *P. hydropathica* isolates were cultivated separately with A1 and A2 mating types of *P. nicotianae* at CA. The plugs from each *P. hydropathica* isolate and *P. nicotianae* A1 or A2 were placed at opposite sides in the Petri dish and were incubated at 20 °C for a month.

The mycelial growth rate of the *Phytophthora* isolates was determined by cultivation on CA medium at nine different temperatures ranging from 5 to 40 °C (5, 10, 15, 20, 25, 30, 35, 37 and 40 °C). All cultures were first incubated for 24 h at 20 °C to synchronize the onset of growth. Then, the mycelial growth of each isolate was marked, and three replicate plates were cultivated at the corresponding temperatures. The radial growth rate (mm/d) was calculated per day along two lines intersecting the center of the mycelium after inoculation for 4–7 days, depending on the temperature.

### 2.4. Pathogenicity Tests

The potential of the selected isolates to infect alder was evaluated by using detached leaf bioassay and inoculation of *A. glutinosa* cuttings. In addition to tests with common alder, pathogenicity analyses with gray alder (*A. incana*) were also performed. Detached leaf bioassay was carried out with young, fully expanded and healthy leaves of both alder species. Mycelial plugs (5 × 5 mm) from the 7-day-old cultures of each isolate were placed on the leaves after slightly wounding the surfaces (two plugs per each leaf). Leaves supplemented with agar plugs without *Phytophthora* were used as a control. The inoculated leaves were incubated onto wet filter paper in the Petri dishes to maintain high relative humidity at 24 °C/20 °C (16 h day/8 h night). Daily monitoring of the inoculated leaves for the appearance of disease symptoms was performed, and the severity of the necroses was evaluated 7 days after inoculation (dpi).

The ability of the studied *Phytophthora* isolates to infect alder cuttings was also evaluated. Young cuttings (6–7 cm) from *A. glutinosa* and *A. incana* were placed separately into plastic trays on wet paper. Mycelial plugs (3 × 3 mm) from the 7-day-old cultures of each isolate were placed on the middle part of the cuttings after slightly injuring the surface layer. The plastic trays were covered to maintain high humidity and were incubated at 24/20 °C (16 h day/8 h night). The disease symptoms were evaluated 4 days after the inoculation of the cuttings.

The statistical significance of the differences between the values in the conducted pathogenicity tests was assessed by *t*-test one-way ANOVA at the probability level *p* ≤ 0.05.

## 3. Results

### 3.1. Isolation and Identification of Phytophthora Species

**Figure 1:**
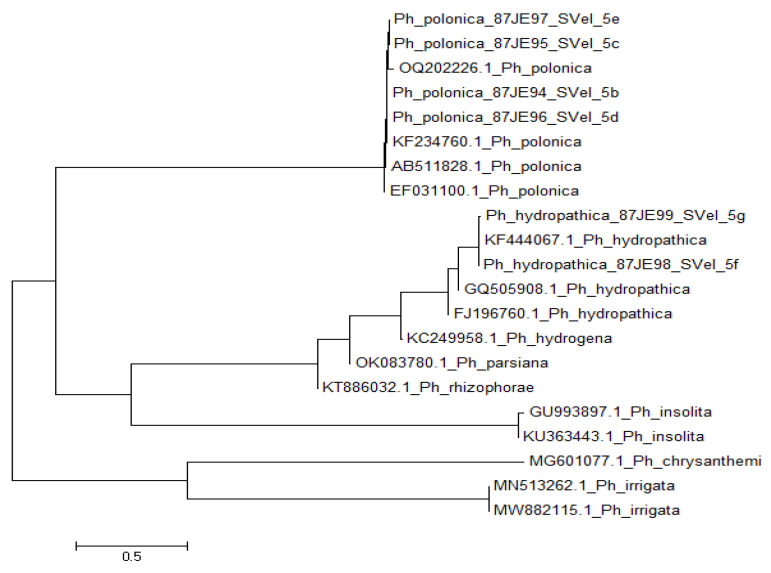
Phylogenetic trees of the isolates *P. polonica* (SVel2022/5b, SVel2022/5c, SVel2022/5d and SVel2022/5e), *P. hydropathica* (SVel2022/5f and SVel2022/5g) and other representatives of clade 9, constructed based on ITS sequencing.

**Figure 2:**
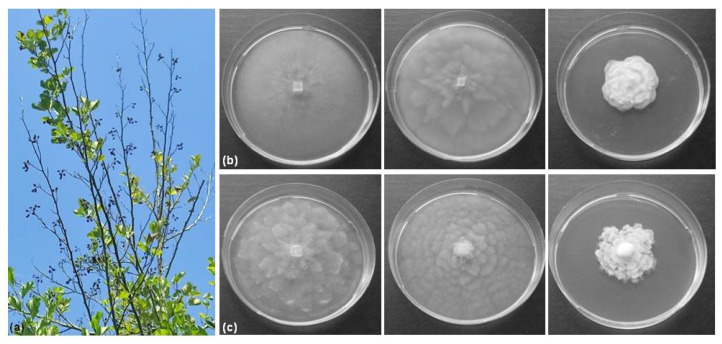
Disease symptoms on an *A. glutinosa* tree and isolation of the causal agents. (**a**) Crown defoliation and branches dieback on common alder; (**b**,**c**) colony morphology of *P. polonica*, isolate SVel5b, *P. hydropathica* and isolate SVel5f, respectively, after 7 days on CA, V8A and PDA (from left to right).

Two *Phytophthora* species were isolated from rhizosphere soil samples collected from *A. glutinosa* trees with disease symptoms in the autumn of 2022. They were identified based on molecular DNA analyses and the morphological characterization of the colony (Figure 1 and Figure 2).

The sequencing of the ITS region showed that four of selected isolates (SVel2022/5b, SVel2022/5c, SVel2022/5d and SVel2022/5e) shared the highest homology with *P. polonica*, whereas two other isolates (SVel2022/5f and SVel2022/5g) demonstrated the highest homology with *P. hydropathica*. The isolates SVel2022/5b (GenBank No: PP837394) and SVel2022/5d (GenBank No: PP837396) demonstrated 98% similarity to the *P. polonica* isolates derived from Serbia and Portugal (KF234760; OQ202226.1) [35,36], whereas the isolates SVel2022/5c and SVel2022/5e shared 97% similarity to the same isolates. Both *P. hydropathica* isolates, SVel2022/5f (GenBank No: PP837391) and SVel2022/5g (GenBank No: PP837392) showed 99% similarity to the isolate *P. hydropathica* from USA (KF444067.1), according to the BLAST search in the NCBI database. Both species, *P. polonica* and *P. hydropathica,* belong to clade 9 of the genus *Phytophthora*. The phylogenetic trees of the studied isolates and several other members of the clades 9, constructed based on ITS sequencing, are presented on Figure 2. This is the first report for the occurrence of *P. polonica* in Bulgaria, according to the literature reference made for the species.

The *P. polonica* isolates were derived from the baits of *Rhododendron* (SVel2022/5b and SVel2022/5c) and oleander (SVel2022/5d and SVel2022/5e), whereas both *P. hydropathica* isolates (SVel2022/5f and SVel2022/5g) originated from the oak baits. The incubation of selected isolates on CA, V8A and PDA at 20 °C revealed fast growth on the first two media by both species, whereas the potato agar was not so suitable for their development (Figure 2b,c). The isolated *P. polonica* isolates formed submerged colonies with stellate to rosaceous mycelia on CA and V8A media. On PDA, the isolates exhibited aerial mycelium growth and rosaceous colonies (Figure 2b). The studied *P. hydropathica* isolates showed petaloid patterns of colonies with aerial growth on all tested media (Figure 2c).

### 3.2. Morphological Characteristics and Radial Growth Rate

**Figure 3:**
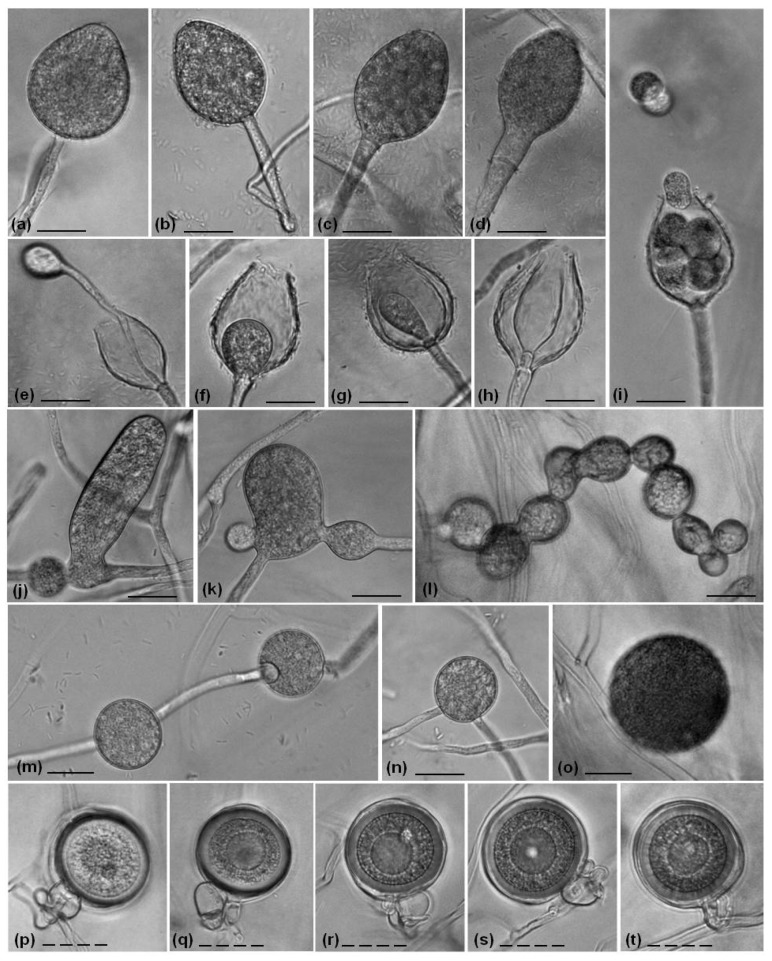
Morphological structures of *P. polonica* isolates observed on V8A (**a**–**o**) and CA (**p**–**t**). (**a**,**b**) Ovoid sporangia of isolates SVel5c and SVel5b, respectively; ovoid sporangia with tapering base of SVel5b (**c**,**d**); (**e**) internal and (**f**–**h**) nested proliferation; (**i**) release of the zoospores of SVel5b; (**j**–**l**) hyphal swelling of SVel5b; (**m**,**l**) chlamydospores of SVel5d and (**o**) SVel5b; (**p**) oogonia with an amphigynous antheridium and (**q**–**t**) paragynous antheridium of SVel5b, SVel5c and SVel5d, respectively. Bars: 20 μm (solid line); 100 μm (dashed line).

The formation of sporangia by *P. polonica* isolates was difficult to perform in a culture medium, and therefore, a special protocol was applied. In contrast, both *P. hydropathica* isolates produced sporangia abundantly. *P. polonica* is characterized by non-papillate ovoid sporangia (Figure 3a,b), sometimes with a tapering base (Figure 3c,d). It proliferated internally (Figure 3e) and nested (Figure 3f–h). The release of zoospores by the isolates was also observed (Figure 3i).

**Figure 4:**
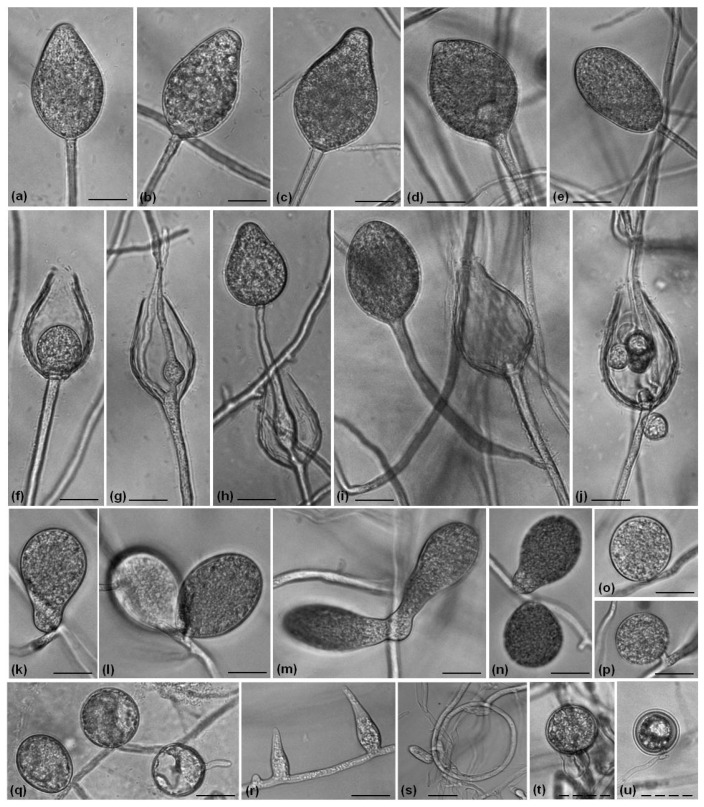
Morphological structures of *P. hydropathica* observed on V8A (**a**–**j**) and CA (**k**–**u**). (**a**–**e**) Sporangia with different shapes of SVel5g: (**a**–**c**) obpyriform, (**d**) ovoid and (**e**) ellipsoid; (**f**) nested, (**g**,**h**) internal and (**i**) external proliferation of Vel5f; (**j**) release of the zoospores of Vel5g; (**k**–**q**) chlamydospores of Vel5g and Vel5f; (**r**) hyphal swelling and (**s**) coiling hyphae of Vel5f; and (**t**,**u**) oogonia of Vel5f. Bars: 20 μm (solid line); 100 μm (dashed line).

The *P. hydropathica* isolates produced non-papillate sporangia too, but they are characterized by a wide variety of shapes, mainly obpyriform (Figure 4a–c) and sometimes ovoid (Figure 4d) and ellipsoid (Figure 4e). The nested (Figure 4f), internal (Figure 4g,h) and external proliferation (Figure 4i) of *P. hydropathica* isolates were documented. The release of zoospores is presented in Figure 4j. Other morphological structures such as chlamydospores (Figure 3m,l and Figure 4k–q) and hyphal swelling (Figure 3j–l and Figure 4r) for both species were monitored, as well as the coiling hyphae of *P. hydropathica* (Figure 4s).

The spontaneous formation of oogonia by all four *P. polonica* isolates on CA media after cultivation for 9 days was observed (Figure 3p–t). They are spherical to subglobose, with thin or smooth walls, predominantly with paragynous antheridium (Figure 3q–t) and occasionally amphigynous antheridium (Figure 3p). As *P. hydropathica* is a heterotallic species, cultivation with different mating types of *P. nicotianae* was performed, and the formation of oogonia was observed after pairing with the A2 type for 3 weeks (Figure 4t,u), which showed that both studied *P. hydropathica* isolates were A1 mating types.

**Figure 5:**
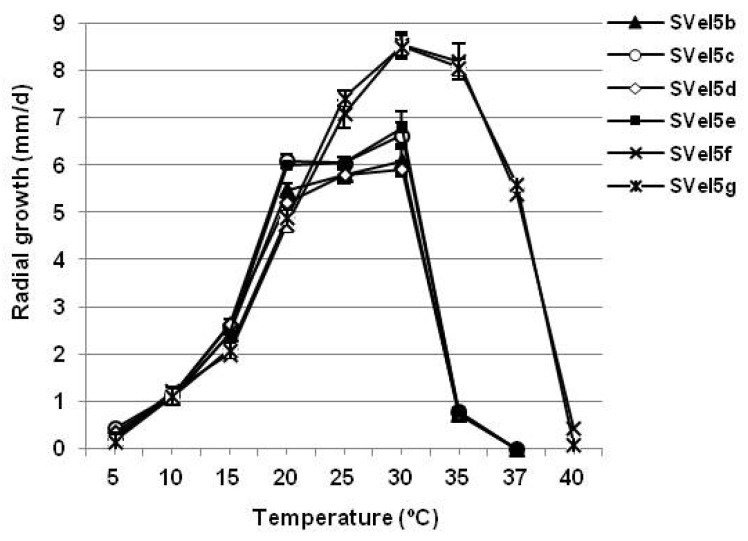
Radial mycelium growth rate of *P. polonica*, isolates SVel5b, SVel5c, SVel5d and SVel5e, and *P. hydropathica*, isolates SVel5f and SVel5g, on CA in the temperature range of 5–40 °C. The bars represent the standard errors of the mean for each temperature.

The cultivation of the studied *P. polonica* and *P. hydropathica* isolates on a CA medium at nine different temperatures in a scale from 5 to 40 °C confirmed the tolerance of both species to high temperatures (Figure 5). The optimum temperature for all isolates was 30 °C, with a daily growth rate of about 6 mm/d for isolates SVel5b and SVel5d, 6.7 mm/d for isolates SVel5c and SVel5e of the species *P. polonica* and reaching 8.5 mm/d for the isolates SVel5f and SVel5g of the species *P. hydropathica*. The maximum temperature for the growth of *P. polonica* was 35 °C (0.8 mm/d), while no mycelial growth of the isolates at 37 °C was observed. The inhibition was reversible for isolates SVel5b, SVel5c and SVel5d after transferring the cultures for cultivation at 20 °C for 10 days, but not for the isolate SVel5e. In contrast, *P. hydropathica* continued to demonstrate very intensive growth at 37 °C with a daily rate of about 5.5 mm/d. However, the mycelial growth of both isolates was sharply suppressed at 40 °C. Incubation at this temperature for 4 days led to the irreversible inhibition of isolate SVel5g, whereas the mycelial growth of isolate SVel5f was restored after one week of cultivation at 25 °C.

### 3.3. Pathogenicity Tests

**Figure 6:**
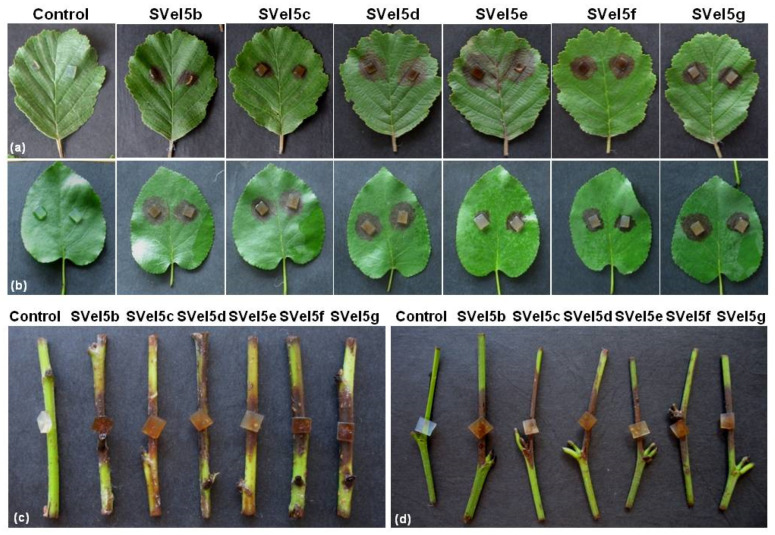
Pathogenicity of the tested isolates of *P. polonica* (SVel5b, SVel5c, SVel5d and SVel5e) and *P. hydropathica* (SVel5f and SVel5g). (**a**,**b**) Disease symptoms on the leaves of *A. glutinosa* and *A. incana*, and (**c**,**d**) on cuttings of both plants species, respectively.

The ability of the selected *P. polonica* and *P. hydropathica* isolates to infect the leaves and cuttings of *A. glutinosa* and *A. incana* is presented in Figure 6 and Table 1. The appearance of disease symptoms 2 days post-inoculation of the cuttings was monitored followed by the infection of the detached leaves at 4 dpi. The development of necrotic lesions was observed on all tested leaves and cuttings of both plant species. The results from the pathogenicity tests were counted at 7 dpi for the detached leaves (Figure 6a,b; Table 1) and at 4 dpi for the cuttings (Figure 6c,d; Table 1). No significant difference in the aggressiveness of the studied isolates from either pathogen was observed. No disease symptoms on the control leaves and cuttings were detected. The re-isolation of the *Phytophthora* species from the necrotic lesions on the infected cuttings and leaves of black and gray alder was performed, according to Koch’s postulates. The experimental data showed that *P. polonica* and *P. hydropathica* can infect not only *A. glutinosa*, the species from which they were isolated, but also *A. incana*.

## 4. Discussion

The species *Phytophthora polonica* was isolated for the first time in Poland [9], and after that, it was identified in several European counties such as Hungary [37], Serbia [35], Ukraine [28], Austria [38] and Portugal [36]. Therefore, it is assumed that the species is native to Europe [38]. In Bulgaria, *P. polonica* was found in the southeastern part of the country, just 1 km from the Black Sea and only about 10 km from the border of Turkey. The region is characterized by broadleaf forests and a transitional Mediterranean climate that is strongly influenced by the sea, which is unique for this part of the country. This is the first report of *P. polonica* in Bulgaria and the most southeastern site where the species has been detected in Europe.

*Phytophthora polonica* has been isolated from rhizosphere soil and is mainly associated with alder decline [2,9,28,38,39], but it is also related to the declining of *Q. robur* trees [40], the dieback of wild cherry trees [37] and the declining of poplar plantations [35]. The species was derived from water streams in two sites in Portugal, too [36]; however, it was not detected at the time of a large-scale investigation of 37 rivers in Austria [38]. Recently, a survey of *Phytophthora* diversity in watercourses in Switzerland resulted in the first report of *P. polonica* and *P. hydropathica* in the country [41]. The isolation of four *P. polonica* isolates from the rhizosphere soil samples of declining alder in Bulgaria confirms the relationship between the dieback of *A. glutinosa* and the pathogen.

*Phytophthora hydropathica* is known as a pathogenic species related to water ecosystems such as rivers, streams and irrigation waters, as well as horticultural crops [10,12,30,42]. The species was associated with alder decline in riparian woodlands for the first time in northwest Spain [30]. It was recently reported as one of the four most frequently isolated species along the black alder waterway in Italy [27]. In this study, *P. hydropathica* was isolated from water and the rhizosphere soil samples of *A. glutinosa*, but not from the rhizosphere of *A. incana*. In contrast to Spain and Italy, extensive investigations of declining alder trees in Ukraine, Portugal and Turkey failed to identify *P. hydropathica* among causal disease agents [28,36,43]. In Bulgaria, it was previously isolated from the Tundzha River and was not associated with any plant host species until now [44].

The results of the pathogenicity tests with the studied *P. polonica* and *P. hydropathica* isolates proved the aggressiveness of both species to the leaves and cuttings of *A. glutinosa*. In addition, their ability to induce disease symptoms on the leaves and cuttings of *A. incana* was demonstrated. The aggressiveness of *P. polonica* and *P. hydropathica* to both plant species is similar, suggesting that gray alder is also at risk from the disease, although the two pathogens originated from the common alder.

The characterization of *P. polonica* and *P. hydropathica* revealed that they have optimum growth rates at 30 °C, which confirms the tolerance of both species to high temperatures. This is in correlation with the unique capacities of most species from clade 9 for adaptation to elevated temperatures [11]. Although *P. polonica* and *P. hydropathica* were inhibited during incubation at 37 °C and 40 °C, respectively, four of the six tested six isolates managed to restore their mycelial growth in a few days after transfer at 20–25 °C. The reversible growth of both species indicates that they could even adapt to increasing average annual temperatures. This advantage will help *P. polonica* and *P. hydropathica* expand their habitats into new areas, and they could be able to find new host species from forest and agricultural ecosystems.

While a number of *Phytophthora* pathogens associated with alder decline in Europe are reported, there is no information about the isolation of *P. polonica* and *P. hydropathica* together. The common effect of these two pathogens on the spread of disease symptoms on alder trees, as well as on the whole ecosystem, is unclear. Therefore, additional research and continued monitoring of the area where both *Phytophthora* species were found are needed to elucidate their long-term impacts on the vegetation in the region.

## 5. Conclusions

The isolation and characterization of *P. polonica* and *P. hydropathica* in southeast Bulgaria are presented in this study. Both species belong to clade 9 of the genus *Phytophthora* and demonstrated typical traits for the corresponding species’ morphological and physiological features. Their pathogenicity to common and gray alder was proved as a result of the conducted experiments. The detection of these plant pathogens in poorly studied areas in Europe, such as Bulgaria, supports the monitoring of the *Phytophthora* distribution and its invasion on new territories. The detailed survey, characterization and investigation of novel hosts can be useful tools for the rapid and accurate recognition of damages caused by these phytopathogens on ecosystems.

## Figures and Tables

**Table 1:** Lesion sizes on leaves and cuttings of *A. glutinosa* and *A. incana* after inoculation with *P. polonica* and *P. hydropathica.*

Isolate	Lesion Size on *A. glutinosa* (mm)		Lesion Size on *A. incana* (mm)	
	Leaves	Cuttings	Leaves	Cuttings
Control	0	0	0	0
SVel5b	13.3 ± 2.3	30.8 ± 6.6	11.6 ± 0.9	23.7 ± 2.3
SVel5c	9.3 ± 1.5	28.9 ± 6.9	11.5 ± 1.3	25.5 ± 5.8
SVel5d	11.5 ± 2.0	24.3 ± 7.1	12.1 ± 1.3	21.8 ± 5.3
SVel5e	12.5 ± 2.3	27.0 ± 6.7	11.3 ± 1.0	17.8 ± 3.1
SVel5f	13.1 ± 1.4	26.3 ± 4.1	13.2 ± 1.2	18.2 ± 4.7
SVel5g	13.0 ± 1.2	31.3 ± 4.6	13.7 ± 1.7	23.3 ± 4.5

## Data Availability

Data are contained within the article.
